# Depression

**DOI:** 10.1186/1472-6874-4-S1-S19

**Published:** 2004-08-25

**Authors:** Donna E Stewart, Enza Gucciardi, Sherry L Grace

**Affiliations:** 1 University Health Network Women's Health Program, University of Toronto, 657 University Ave, ML-2004, Toronto, M5B 2N2, ON, Toronto, Canada; 2 University Health Network Women's Health Program, University of Toronto, 657 University Ave, ML-2004, Toronto, M5B 2N2, ON, Toronto, Canada; 3 University Health Network Women's Health Program, University of Toronto, 657 University Ave, ML-2004, Toronto, M5B 2N2, ON, Toronto, Canada

## Abstract

**Health Issue:**

Depression causes significant distress or impairment in physical, social, occupational and other key areas of functioning. Women are approximately twice as likely as men to experience depression. Psychosocial factors likely mediate the risks for depression incurred by biological influences.

**Key Findings:**

Data from the 1999 National Population Health Survey show that depression is more common among Canadian women, with an annual self-reported incidence of 5.7% compared with 2.9% in men. The highest rates of depression are seen among women of reproductive age. Predictive factors for depression include previous depression, feeling out of control or overwhelmed, chronic health problems, traumatic events in childhood or young adulthood, lack of emotional support, lone parenthood, and low sense of mastery. Although depression is treatable, only 43% of depressed women had consulted a health professional in 1998/99 and only 32.4% were taking antidepressant medication. People with lower education, inadequate income, and fewer contacts with a health professional were less likely to receive depression treatment.

**Data Gaps and Recommendations:**

A better understanding of factors that increase vulnerability and resilience to depression is needed. There is also a need for the collection and analysis of data pertaining to: prevalence of clinical anxiety; the prevalence of depression band 12 months after childbirth factors contributing to suicide contemplation and attempts among adolescent girls, current treatments for depression and their efficacy in depressed women at different life stages; interprovincial variation in depression rates and hospitalizations and the impact and costs of depression on work, family, individuals, and society.

## Background

Major depression is a common, disabling disorder characterized by a period of at least two weeks in which a person loses pleasure in nearly all activities and/or exhibits a depressed mood. Symptoms of major depression include feelings of sadness and hopelessness, diminished interest and pleasure, changes in weight and in sleep patterns, chronic fatigue, feelings of worthlessness or guilt and difficulty concentrating or thinking. These symptoms cause clinically significant distress or impairment in physical, social, occupational and other key areas of functioning. [1]

### Differences between Men and Women

Women do not experience more mental illness than men; they are simply more prone to depression and anxiety, whereas men are more likely to have addictive disorders and personality disorders. [2] Women are approximately twice as likely as men to experience a depressive episode within a lifetime. Sex differences in rates of depression emerge at puberty and decline after menopause, highlighting the complex and reciprocal interactions that occur between biological, psychological and sociocultural factors. [2] It is likely that psychosocial factors mediate the risks incurred through biological influences, such as a disturbance in the interaction between the hypothalamic-pituitary-gonadal axis and neuromodulators. The effects of stress, violence, poverty, inequality, sexism, caregiving, relational problems, low self-esteem and ruminative cognitive styles probably increase vulnerability to depression in women. [3]

Women are more likely to present with "atypical" depression, including mood reactivity (mood that brightens in response to actual or potentially positive events), significant weight gain or increase in appetite, hypersomnia, heavy leaden feelings in the arms or legs, and long-standing patterns of sensitivity to interpersonal rejection. [1] A subgroup of women may be especially sensitive to normal physiological hormonal variations and may present with depression premenstrually, in the postpartum period or during perimenopause. [4]

### International Trends

Recent studies suggest that the prevalence rates of depression and anxiety are increasing worldwide. [5,6] For example, a cohort analysis from the National Cohort Morbidity Study in the United States revealed that the lifetime prevalence of depression among women aged 20 to 24 increased from 6% in the early 1960s to about 28% in the early 1990s. This is probably evidence of the influence of a changing environment on depressive symptoms, as neither the gene pool nor the distribution of sex hormones could have changed significantly during that period. [7]

Results from the Global Burden of Disease Study, a collaborative effort by the World Health Organization, World Bank and Harvard School of Public Health, showed that mental disorders contribute more to the global burden of disease than all cancers combined. [8] The lifetime rate of major depressive episodes among Canadian women (12.3/100) falls between rates reported in the United States (approximately 7.5/100) and France (21.9/100). [9]

### Canadian Trends

**Figure 1:**
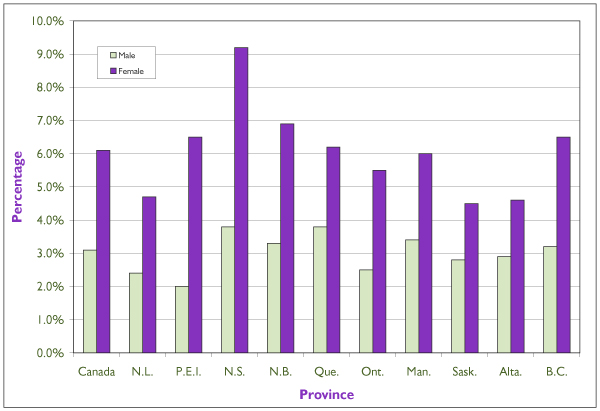
**Prevalence of depression by sex and province, 1998–1999 **Source: National Population Health Survey, 1998–1999

**Figure 2:**
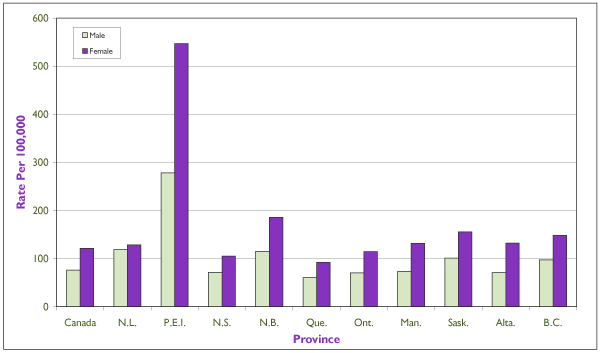
**Hospital admissions for depression, by sex and province (age ≥ 15 years), 1999 **Source: Hospital Morbidity Database, 1998–1999

There are significant interprovincial variations in the prevalence of depression in Canada, as shown in the NPHS 1998–1999. In all provinces, the proportion of women with depression in 1998–1999 was much greater than that of men. For women the highest proportions were in Nova Scotia and New Brunswick and the lowest in Saskatchewan and Alberta (Figure 1). There are also significant interprovincial variations in hospitalization for depression, Prince Edward Island showing the highest and Quebec the lowest rates among women (Figure 2). The atypically high rates of hospitalization for depression in Prince Edward Island appear to result from unusual practice or coding patterns, as the reported rates of depression and attempted suicide are not significantly different from those of other provinces.

### Suicidal Behaviours

**Figure 3:**
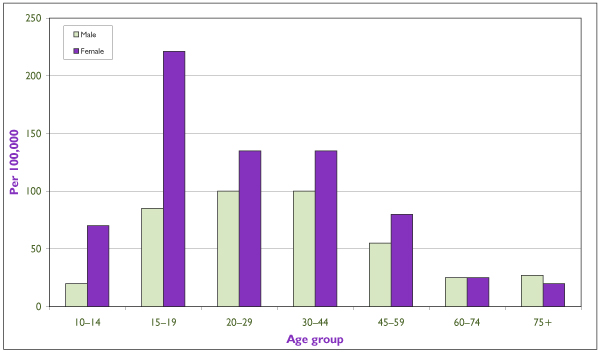
**Age-specific hospitalization rates for suicide attempts, by sex, Canada, 1998–1999 **Source: Hospital Morbidity Database, 1998–1999 (*Health Rep *2002;13(2))

**Figure 4:**
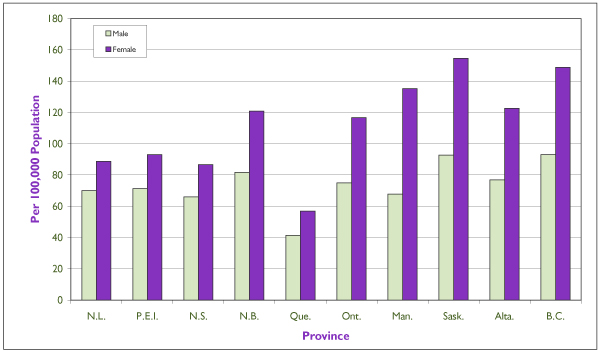
Age-standardized hospitalization rates for suicide attempts,* by sex and province, 1998 Source: Hospital Morbidity Database, 1998–1999 (*Health Rep *2002;13(2))

Attempted suicide rates are difficult to gauge, but the Canadian age-standardized hospitalization rate among people aged 10 years and older was 108 per 100,000 females and 70 per 100,000 males in 1998. [10] The highest rates are found among female adolescents aged 15 to 19 years (220.8 per 100,000). Age-specific hospitalization rates for suicide attempts are shown in Figure 3. These rates vary significantly among territories and provinces: the Northwest Territories, Yukon, Saskatchewan and British Columbia have the highest age-standardized hospitalization rates for suicide attempts, and Quebec and Nova Scotia the lowest among men and women (Figure 4). In the National Longitudinal Survey of Children and Youth (1996–1997) it was found that girls are more likely than boys to report suicidal thoughts. [10]

**Figure 5:**
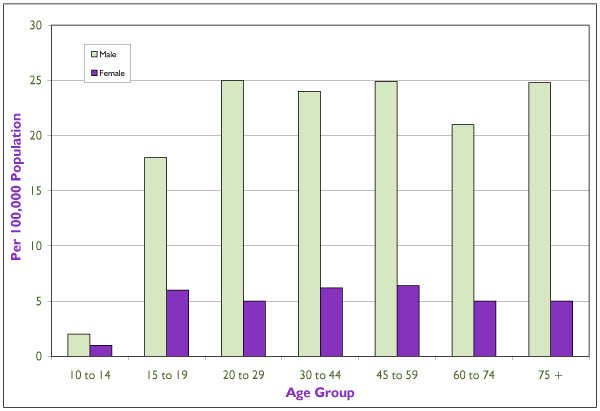
**Age-specific suicide rates, by sex, Canada, 1998 **Source: Canadian Vital Statistics Database (*Health Rep *2002;13(2))

**Figure 6:**
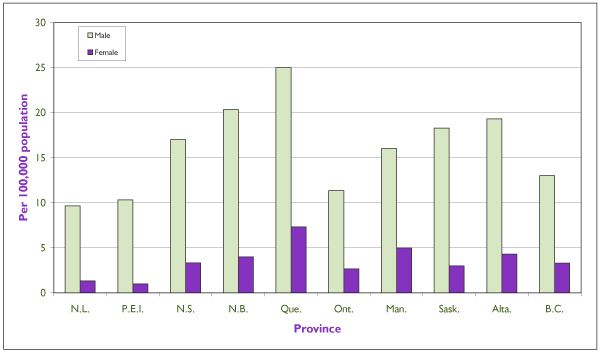
Age-standardized suicide rates,* by sex and province, 1998 Source: Canadian Vital Statistics Database, 1998 (*Health Rep *2002;13(2))

Suicide rates are sometimes used as a proxy for mortality in depression, but suicide is associated with many other factors, such as physical illness, substance abuse, family violence and social isolation. [11] In 1998, the rate in Canada was 14 suicides per 100,000 population. Suicide was the leading cause of death among men aged 25 to 29 and 40 to 44, and among women aged 30 to 34 years. [11] The overrepresentation of men in deaths by suicide is consistent across all Western countries. Figure 5 shows the age-specific suicide rates in Canada. Quebec has a significantly higher rate of suicide, and the Maritime provinces, Ontario, Saskatchewan and British Columbia have the lowest rates among women (Figure 6).

### Vulnerable Subgroups

Depression appears to be disproportionately high among Aboriginal populations in Canada, and up to age 65 the suicide rate in this population is higher than that among other Canadians. Suicide rates increase over the teenage years and peak at age 23 to 25. Among women, status Indian adolescents are 7.5 times as likely as other Canadian adolescents to commit suicide, and, between 20 and 29 years of age, status Indian women have a suicide rate 3.6 times that of other similarly aged Canadian women. Attempted suicide rates are also higher in Aboriginal populations, in which suicide attempt clusters pose special problems. [12]

We were unable to find sources for national representative data on depression in diverse ethnic or cultural groups, immigrants, lesbian or transsexual women.

### Current Study

The information below results from analyses of data from the NPHS with specific regard to the prevalence of depression in Canada, possible risk factors and therapeutic interventions.

## Methods

The NPHS has a longitudinal survey component that collects information from the same panel of respondents every two years (see Appendix A for details about the methods of data collection for the NPHS). Examination of data from the first three cycles (1994–1995, 1996–1997, 1998–1999) allows an exploration of changes in prevalence over time and an assessment of the importance of particular social or psychological factors in predicting depression. The NPHS measures major depressive episodes (MDE) with a subset of questions from the Composite International Diagnostic Interview, which covers a cluster of symptoms listed for depression in the *Diagnostic and Statistical Manual of Mental Disorders *[1] version III-R.

## Results

### Prevalence

**Figure 7:**
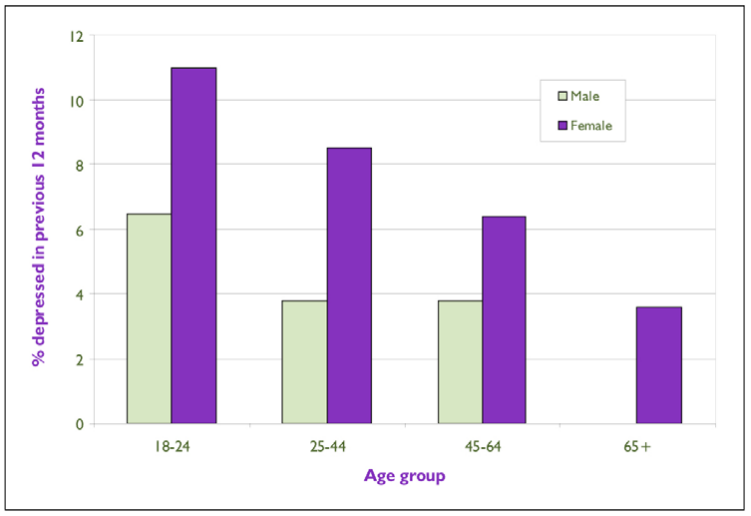
**Unadjusted prevalence of depression, by sex and age group, Canada, 1994–1995: proportion depressed in previous 12 months. **Source: National Population Health Survey, 1994–1995 (*Health Rep *1996;7(4)).

Information from the first three cycles of the NPHS on Canadians aged 12 and over indicated that approximately twice as many women as men reported symptoms of a major depressive episode: in 1994–1995, 7.1% of women compared with 3.3% of men; in 1996–1997, 5.4% of women compared with 2.7% of men; and in 1998–1999, 5.7% compared with 2.9%. The prevalence of depression was higher among women between the ages of 18 and 24 than among older women. At age 45 and over, the rates of depression among women began to fall, and by age 65 they approximated those of men (3.1%) (Figure 7). [13]

### Risk Factors

#### Social and Psychological Factors

**Figure 8:**
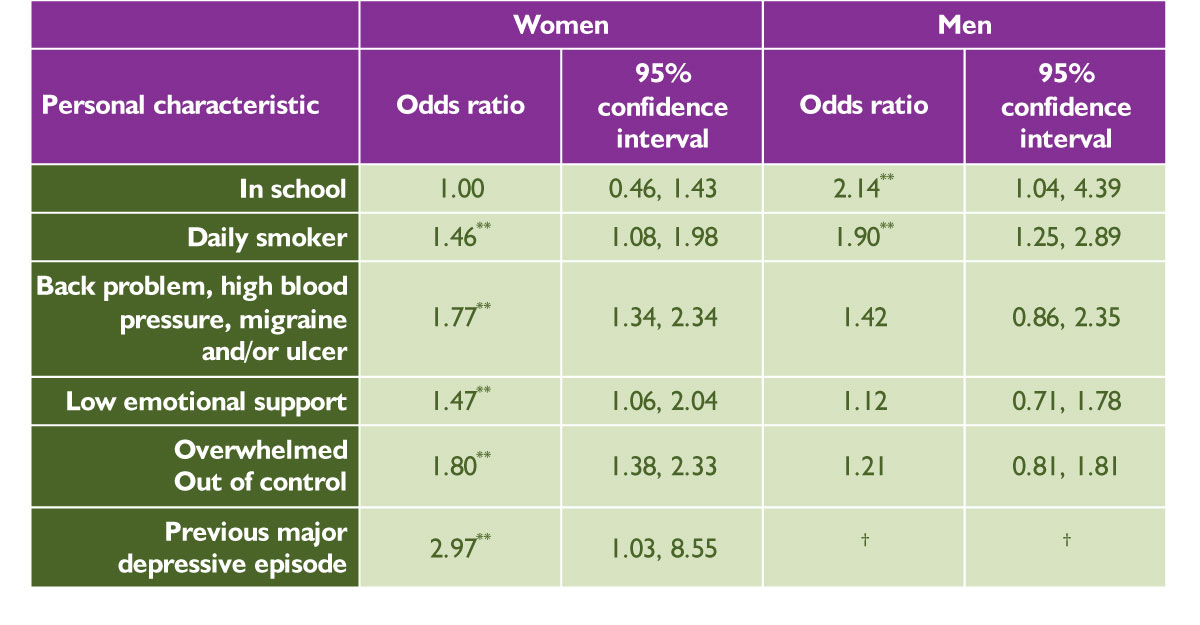
Adjusted odds ratio of a depressive episode* in 1996–1997 and/or 1998–1999, by selected characteristics in 1994–1995, Canada excluding territories Source: National Population Health Survey, longitudinal file, 1994–1995 to 1998–1999 (*Health Rep *1999;11(3))

Psychosocial factors from the first NPHS wave (1994–1995) were tested to predict depression in the next two waves (Figure 8). [11] Neither educational attainment, employment/main activity nor low income was predictive of depression in general. [11] Among women, three measures were significantly associated with a later major depressive episode: previous major depressive episode (odds ratio [OR] 2.97), feelings of being overwhelmed and out of control (OR 1.80) and lack of emotional support (OR 1.47). [11] Similarly, women who lacked emotional support in the 1994–1995 NPHS had higher odds of future depressive episodes than women who had adequate social support, illustrating the critical role of social buffering. [14] The diagnosis of a chronic health problem was also predictive of depression in women only (OR 1.77). Some factors differentially increased the risk of depression in men and women. For example, traumatic events in childhood or young adulthood (OR 1.69) and a low sense of mastery (OR 1.32) were associated with a high risk of depression for women but not for men. [13]

#### Marital Status

Married status is a greater buffer against depression in men than in women, as shown by 1994–1995 NPHS data. For men, never having been married (OR 2.03) and having previously been married (OR 3.53) heightened the risk of depression. [13] Married people in general are less at risk of depression than singles, although the direction of the relation is unclear: it could be that happy people are more likely to find and keep partners or that the support of marriage provides a protective effect.

Lone parenthood is associated with depression in women, as 15% of lone parent women had experienced a major depressive episode in the previous 12 months as compared with 7% of other women. [13]

#### Childbirth

From the 1998–1999 NPHS data we found lower rates of depression among women who had delivered a child within the previous two years (4.6%) than among women who had not (7.2%). It may be that having a child under 2 years is protective against depression, especially in a supportive environment. Better data (within 12 months after childbirth) are needed to further evaluate this finding, as the current data include women outside the reproductive age range.

#### Education

The 1996–1997 cycle of the NPHS showed a statistically non-significant greater incidence of depression over three years among women with less than secondary education (5.0%) as compared with those with post-secondary education (3.5%). [11] Although education does not appear to protect against depression, it gives women more options in life and work, and permits them to become better informed about depression and ways to access services and treatment.

#### Employment or Main Activity

Employment was not associated with increased odds of having a major depressive episode for men or women, although men who were in school had twice the odds of a future depressive episode than men who were working. [11] Among women, there was a significantly lower two-year incidence of depression (between first and third NPHS waves) among those who were retired (1.8%) than among those who worked (4.5%). [11] This may relate to the "double shift" experienced by many women who provide homemaking and child care as well as work outside the home.

#### Income

In the 1994–1995 NPHS data, among men in the lowest household income quartile the prevalence of depression was twice that of men in higher income quartiles, but the relative difference was smaller among women. Approximately 10% of women in the lowest household income quartile, as compared with 7% of women in the highest quartile, reported depression. [13] Although there are certainly stresses associated with low income, it may be that women's self-esteem is less directly linked to income than men's.

#### Physical Health

Individuals who had had a depressive episode in the year before their baseline 1994–1995 NPHS interview had nearly three times the odds (OR 2.7, 95%, confidence interval [CI] 1.01, 7.04) of being given a diagnosis of heart disease in the subsequent five years as individuals who had not experienced a depressive episode during that time. [15] The relation of depression to a host of chronic diseases is a subject of ongoing investigation. In addition, it is well known that physical symptoms such as fatigue, pain, and sleep and appetite disturbances are common symptoms of depression. [1] According to 1998–1999 NPHS data, a diagnosis of chronic health problems is predictive of depression for women only. [11]

#### Comorbidity

At least 50% of patients with a primary diagnosis of depression or an anxiety disorder have another associated psychiatric disorder, [6] the most common being anxiety disorders, eating disorders and post-traumatic stress disorder, all of which are much more common among women than men. Other common psychiatric comorbidities with depression are substance abuse disorder and some personality disorders. [6]

#### Pain

We were interested in the effects of pain severity on depression in women and men in the 1998–1999 NPHS. Among men with mild, moderate and severe pain or discomfort, 5.12%, 7.60% and 12.3% respectively reported depression; among women the proportions were 7.40%, 11.15% and 16.46%. Greater severity of pain clearly is associated with greater prevalence of depression among both women and men.

#### Weight

In the 1998–1999 NPHS we found that women with a body mass index (BMI) under 20 were the least depressed (mean = 0.39) whereas those with a BMI of 25–27 (overweight) were most depressed (mean = 0.63). The opposite relation between weight and depression was found in men: those with a BMI under 20 were most depressed (mean = 0.49), and those with a BMI of 25–27 were least depressed (mean = 0.20). This discrepancy possibly relates to societal role values that favour slim women and muscular men.

#### Smoking

Nicotine is thought to have antidepressant effects. People who smoked at least daily in 1994–1995 had increased odds of having a major depressive episode as compared with non-smokers in later cycles of the NPHS. These odds were almost double for men and one and a half times higher (OR 1.46) for women who smoked daily. [11] Smokers who have a history of depression have more difficulty quitting smoking and more severe withdrawal symptoms, and they are more susceptible to depression after they stop smoking than smokers who are not prone to depression. [11] Early identification and treatment of depression could play a vital role in smoking prevention, reduction or cessation.

### Interventions

Under-reporting of depression by individuals themselves and by families, friends and health care providers remains an enormous public health problem. A review of published randomized controlled trials in primary care settings shows that screening adults for depression can improve outcomes, particularly when combined with system changes that help ensure adequate treatment and follow-up. [16]

Although the number of women who seek and obtain treatment for depression is greater than that for men, this still represents only a small proportion of depressed women. Data from the 1994–1995 cycle of the NPHS revealed that although depression is amenable to treatment, only 43% of those who met the criteria for major depressive episodes in the previous year reported talking to a health professional about their emotional problems. [17] Treatment for depression is ideally provided through pharmacotherapy, or psychotherapy, or a combination of the two. About 75% of individuals in the 1994–1995 NPHS cycle who suffered from a major depressive episode were below the minimum four visits deemed necessary for acute treatment. [17]

Depressed people whose physical health was good or who had not recently experienced a negative life event were less likely to be treated. According to multivariate analysis, lower education, inadequate income and fewer contacts with a general practitioner reduced the odds of being treated. Also, married people who were depressed were less likely to receive treatment. [17] Lower educational attainment is likely associated with less information about depression, ways to access treatment and the effectiveness of treatment. Such individuals may also be more deterred by social stigma and feel less understood by mental health professionals. Inadequate household income is a barrier to obtaining services outside provincial health care plans, which may not cover psychologist and social worker services. Moreover, income may also restrict the ability to purchase those prescribed medications that are most costly but have fewer side effects, such as the newer antidepressants.

It is clear from all these data that depression is greatly under-treated in Canada. NPHS data in 1998–1999 showed that 83.67% of depressed men and 67.62% of depressed women were not taking antidepressants. With respect to treatment, differences in antidepressant adverse drug reactions have been reported between men and women. [18-20] Other investigators have reported that the average major depressive episode lasts about nine months in the absence of treatment, and about 50% of individuals who have one episode will experience a recurrence. [21] Interpersonal psychotherapy may be more effective than other forms of psychotherapy for women. [21]

## Discussion

### Data Limitations and Gaps

There are currently no Canadian surveillance data on anxiety disorders, which frequently accompany depression, or on violence and discrimination as risk factors for depression in women. There are no available national data on the prevalence of depression in subgroups, including Aboriginal, ethnic minority, lesbian or transsexual women. The impact of women's caregiving role on depression is still unknown as we move to more home care for acute and chronic illness. Data on the use of pharmacotherapy for depression are not available on a national level.

### Surveillance Recommendations

• Commission research to gain a better understanding of the factors that increase or decrease vulnerability and resilience to depression and begin to conduct surveillance on these factors.

• As 8% of girls aged 12 to 13 report having contemplated suicide in the previous year, collect and analyze data to help us understand the contributing factors and ways to improve self-esteem in early adolescent girls.

• Collect and analyze national data on the prevalence of anxiety disorders.

• Collect and analyze data on current treatments and their efficacy in depressed women at different stages of their lives to determine what treatments and services are optimal.

• Conduct further research to better understand interprovincial variations in depression rates and hospitalization rates for women to develop the best possible practices for prevention and treatment strategies.

• Collect and analyze data on the impact of depression on work and family life and the costs to the individual, family and society.

• Collect and analyze data on depression at 6 and 12 months after childbirth to determine its prevalence and the factors that increase or decrease vulnerability to depression.
